# Evaluation of performance and microbial community successional patterns in an integrated OCO reactor under ZnO nanoparticle stress

**DOI:** 10.1039/c8ra05057k

**Published:** 2018-07-30

**Authors:** Zhenghui Liu, Huifang Zhou, Jiefeng Liu, Mei Huang, Xudong Yin, Zhisen Liu, Yufeng Mao, Wenyu Xie, Dehao Li

**Affiliations:** School of Environmental and Biological Engineering, Guangdong University of Petrochemical Technology Maoming Guangdong 525000 China dehlee@163.com; Technology Research Center for Petrochemical Resources Clean Utilization of Guangdong Province Maoming Guangdong 525000 China

## Abstract

An integrated OCO reactor was used to investigate the performance and microbial community successional changes under long-term exposure to relatively low levels of ZnO nanoparticles (NPs). Relatively higher concentrations of ZnO NPs (1.5 mg L^−1^) could adversely affect the nitrogen and phosphorus removal in the reactor. The diversity and richness of the microbial communities chronically declined with an increasing concentration of ZnO NPs higher than 1.5 mg L^−1^. With the elevated ZnO NPs, the phyla abundances of *Proteobacteria*, *Firmicutes* and *Actinobacteria* decreased slightly, whereas those of *Bacteroidetes* and *Acidobacteria* increased. *Bacteroidetes* and *Proteobacteria* were the predominant phyla in each phase (with a variation in abundance), together with some common taxa responses to ZnO NP stress as revealed by Venn diagram analysis. Some genera associated with the removal of nitrogen and phosphorus, such as *Acinetobacter*, *Stenotrophomonas* and *Pseudomonas*, decreased significantly. The present results are significant for expanding our understanding of the functional performance and microbial community successions of activated sludge which has experienced long-term exposure to environmentally relevant concentrations of ZnO NPs.

## Abstract 2

An integrated OCO reactor was used to investigate the performance and microbial community successional changes under a long-term exposure to relatively low-level ZnO nanoparticles (NPs).

## Graphical abstract

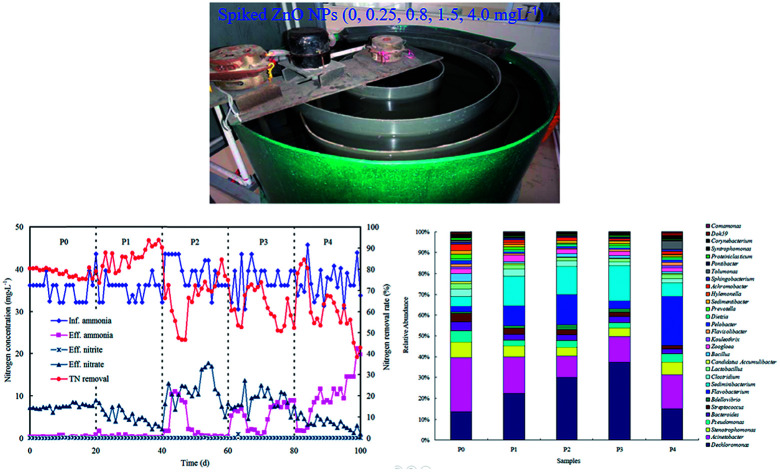

## 1. Introduction

With the rapid development of nanotechnology, the application of engineered nanomaterials (ENMs) in a variety of industrial products and consumer goods has accelerated. Among ENMs, ZnO nanoparticles (ZnO NPs) together with titanium dioxide NPs and silver NPs are the most frequently used inorganic species in these industrial products and consumer goods.^1^ Taking into consideration that NPs might enter into domestic sewage and industrial wastewater systems, their potential effects on the environment and human health have raised increasing concerns.

ZnO NPs are widely used in paints, coatings, cosmetics, photocatalysts, therapeutics, drug delivery systems and semiconductors, due to their antimicrobial, catalytic and ultraviolet-protective properties.^2^ Previous studies reported the occurrence of ZnO NPs in wastewater treatment plants (WWTPs).^3,4^ Considering that activated sludge plays a crucial role in biological wastewater treatment, the interaction of activated sludge with NPs may compromise the performance of WWTPs. Currently, most reports have focused on the toxic effects of ZnO NPs on specific bacteria, such as *Escherichia coli*^5^ and *Pseudomonas stutzeri*.^6^ The impact of ZnO NPs on the microbial communities of the activated sludge has also been investigated recently. Based on high-throughput pyrosequencing, Wang *et al.* reported that the abundance of nitrite oxidizing bacteria (NOB) increased but the abundance of ammonium oxidizing bacteria (AOB) and denitrifying bacteria was relatively stable in a membrane bioreactor (MBR);^7^ Zhang *et al.* reported the toxicity effect of ZnO NPs on nitrogen removal and microbial communities in a CANON reactor.^8^

Although valuable findings have been documented based on current organismal studies, it is necessary to present a comprehensive understanding that microbial communities act as a whole in maintaining the key functions of a complex environment from an ecological perspective. Several studies have focused on the long-term effects of elevated ZnO NP concentrations on the microbial communities in wastewater treatment systems.^8–10^ In these studies, the ZnO NP concentrations used were relatively high (over 5 mg L^−1^), although admittedly, high concentrations of NPs are required to function in traditional toxicity assays,^11^ meanwhile the environmentally relevant concentration of NPs increases inevitably.^9^ Given that the highest concentration of ZnO NPs in WWTPs reaches 300 μg L^−1^,^4^ and 5 mg L^−1^ of ZnO NPs causes no inhibitory effects on the microbial growth,^8^ we hypothesized that relatively lower concentrations of NPs may have an unpredictable influence on the activated sludge systems over time. Research should provide insight into the long-term effects of environmentally relevant concentrations (or higher by about an order of magnitude) of ZnO NPs on the ecological function of microbial communities in activated sludge.

In the present study, activated sludge systems in an integrated OCO reactor (an anaerobic/anoxic/oxic (A^2^/O) process with a shape similar to the letters of the alphabet “OCO”)^12^ were treated with various concentrations of ZnO NPs (0.25 mg L^−1^, 0.8 mg L^−1^, 1.5 mg L^−1^ and 4 mg L^−1^) for a performance period of 80 days, and the shifts in the microbial communities were revealed using Illumina Miseq pyrosequencing of the 16S rRNA gene. The objectives of this study were as follows: (1) to evaluate the ecotoxicity of ZnO NPs on wastewater treatment systems, and (2) to illustrate the responses of microbial communities to the long-term exposure of relatively lower concentrations of ZnO NPs.

## 2. Materials and methods

### 2.1. Experimental set-up and operation

The integrated OCO reactor with an effective volume of 240 L has been operating for more than one year, and the reactor has been described previously.^12^ The synthetic wastewater consisted of glucose, sucrose, soluble starch, NH_4_HCO_3_, KH_2_PO_4_, and a trace element solution (including MgCl_2_, CaCl_2_, MnSO_4_, CoCl_2_, CuSO_4_, FeSO_4_ and H_3_BO_3_) as previously reported.^13^ The parameters COD, TN and TP of the synthetic wastewater were adjusted to about 300 mg L^−1^, 37 mg L^−1^ and 5 mg L^−1^, respectively. The hydraulic retention time was 12 h (1.6 h for the anaerobic zone, 3.3 h for the anoxic zone, 4.8 h for the aerobic zone, and 2.3 h for the mixed zone and sedimentation tanks). The influences of the ZnO NPs on nutrient (nitrogen and phosphorus) removal and on the microbial community were investigated after the reactor reached a relatively steady state.

### 2.2. Nanoparticle and preparation of nanoparticle suspensions

ZnO nanoparticles were purchased from Shanghai Hansi Chemical Industry Co. Ltd (Shanghai, China), and the average diameter of the nanoparticles was about 30 nm. The size and shape of the nanoparticles were also characterized *via* transmission electron microscopy (TEM) images using an FEI Tecnai G20 microscope with a voltage of 200 kV (FEI company, USA). A nanoparticle stock suspension of 100 mg L^−1^ ZnO NPs was concocted by adding 0.1 g ZnO NPs to 1 L Milli-Q water, with sonication (300 W) for 20 min. Then, various concentrations of ZnO NP suspensions were prepared by diluting the stock suspension with Milli-Q water.

### 2.3. Batch experiments with exposure to ZnO NPs

The integrated OCO reactor was initiated with the inoculation of activated sludge from a full-scale wastewater treatment plant in Maoming, Guangdong, China. Prior to the addition of ZnO NP suspensions in the influent, the integrated OCO reactor had operated continuously for 20 d maintaining a steady operation performance. Then, ZnO NP suspensions were freshly prepared and added to the reactor with final concentrations of 0.25 mg L^−1^, 0.8 mg L^−1^, 1.5 mg L^−1^ and 4 mg L^−1^ for continuous treatment, and the duration time of each treatment was 20 d.

### 2.4. Analytical methods

The temperature, dissolved oxygen (DO) and pH values were measured using portable apparatus with specific probes (WTW, Germany). NH_4_^+^–N, NO_2_^−^, NO_3_^−^, the total phosphorus (TP) and the chemical oxygen demand (COD) were measured according to standard methods.^14^ The total nitrogen (TN) was calculated as the sum of NH_4_^+^–N, NO_2_^−^ and NO_3_^−^.

### 2.5. Pyrosequencing and phylogenetic assignment

The total genomic DNA from each sludge sample was extracted and purified using the FastDNA® Spin Kit for Soil (MP-Bio, USA) according to the manufacturer’s instructions. The DNA concentration and quality were detected with 1% agarose gel electrophoresis and a NanoDrop Spectrophotometer. The partial 16S rRNA genes were amplified with barcoded primers 515F (5′-GTGCCAGCMGCCGCGGTAA-3′) and 909R (5′-CCCCGYCAATTCMTTTRAGT-3′), and pyrosequencing was carried out using an Illumina Miseq sequencing platform.^15^ More than 10 000 sequences with a 350 bp length were obtained from each sample. The aligned sequences were used for a chimera check using the Uchime algorithm.^16^ Taxonomic classifications were performed using the Ribosomal Database Project classifier^17^ with a set confidence threshold of 80%, and the operational taxonomic units (OTUs) were defined at a 3% dissimilarity cutoff. The Shannon diversity index, Chao 1 index, ACE index and Simpson index were analyzed by the QIIME software package. The similarities and differences among these communities were analyzed through Venn diagrams with shared and unique OTUs.^18^

## 3. Results and discussion

### 3.1. Effects of ZnO NPs on reactor performance

**Fig. 1:**
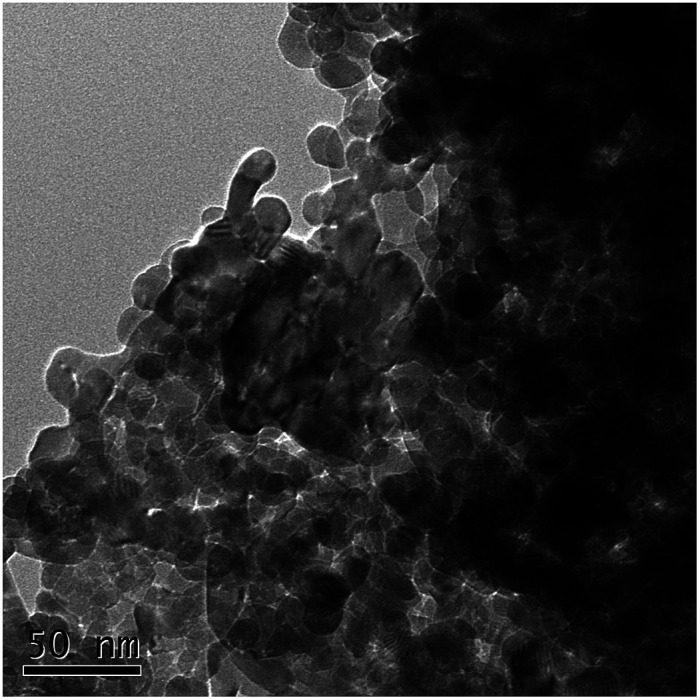
High-resolution TEM image of the ZnO nanoparticles.

**Fig. 2:**
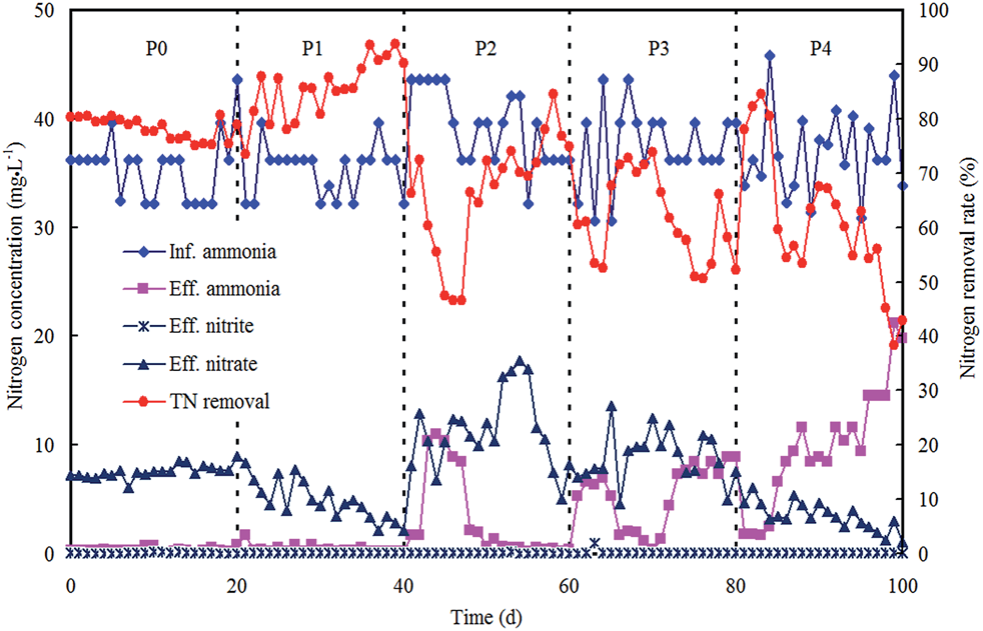
Reactor performance on nitrogen removal in each phase with various concentrations of ZnO NPs.

The morphological characterizations of the ZnO NPs are shown in Fig. 1, and the average diameter of the nanoparticles was 30 nm. The performance of the OCO reactor in each phase has been depicted in Fig. 2. During the whole experiment, the influent concentrations of ammonia and phosphorus were constantly kept around 37 mg L^−1^ and 5 mg L^−1^, respectively. The concentration of the ZnO NPs was increased step-wise during each phase except in phase P0 (which acted as the control).

For nitrogen removal, no nitrite accumulation was observed throughout the dosing phases, indicating that the oxidation of nitrite remained unaffected. The nitrogen removal rate (NRR) reached an average of 0.055 kg m^−3^ d^−1^ (Table 1) in P0 with effluent nitrate at 7.55 mg L^−1^ and a low ammonia level. Then in phase P1, the ZnO NP stock suspension was directly added to the reactor for a concentration of 0.25 mg L^−1^ in the system. The NRR increased and reached an average of 0.060 kg m^−3^ d^−1^ (Table 1), and this result was mainly attributed to the additional removal of nitrate, as Zn^2+^ ions can be released from the ZnO NPs (0.13 mg L^−1^ Zn^2+^ ions within 1 mg L^−1^ ZnO NPs in wastewater),^19^ and low Zn^2+^ concentrations provide essential micronutrients for vital cofactors of metalloproteinases and certain enzymes thereby enhancing the bioactivities of nitrifiers and denitrifiers.^20^ Subsequently, the ZnO NP concentration was further increased to 0.8 mg L^−1^ in phase P2, and the NRR decreased significantly to an average of 0.049 kg m^−3^ d^−1^ (*p* < 0.05); both the ammonia and nitrate in the effluent presented an increasing trend. Then in phase P3, the ZnO NP concentration was further increased to 1.5 mg L^−1^ and the NRR exhibited a small decrease (0.047 kg m^−3^ d^−1^). This indicated that nitrifiers were significantly suppressed. Finally, in phase P4, as the ZnO NP concentration was increased to 4 mg L^−1^, the NRR remained almost unchanged (0.047 kg m^−3^ d^−1^), meanwhile with a decline in the effluent nitrate and a remarkable increase in the effluent ammonia, the ammonia removal rate had dramatically decreased to 41.48% by the last day. The decrease in the ammonia removal rate may be due to a long-lasting inhibition of nitrification, which was strengthened by the accumulative effect of the ZnO NPs. These results coincided with previous studies which proved that ammonia removal is inhibited by inhibition of the respiration of nitrifying bacteria^21^ or enzyme activity.^22^

**Table 1:** Operational conditions and nutrient removal performances (average data of each phase)

Phase	Period (d)	ZnO NPs (mg L^−1^)	Inf. NH_4_^+^ (mg L^−1^)	NRR (kg m^−3^ d^−1^)	ΔNO_3_^−^/ΔNH_4_^+^	MLVSS (mg L^−1^)
P0	0–20	0	35.5	0.055	0.217	2016
P1	21–40	0.25	35.2	0.060	0.141	2163
P2	41–60	0.8	39.3	0.049	0.316	1807
P3	61–80	1.5	37.5	0.047	0.285	1579
P4	81–100	4.0	36.8	0.047	0.127	1261

**Fig. 3:**
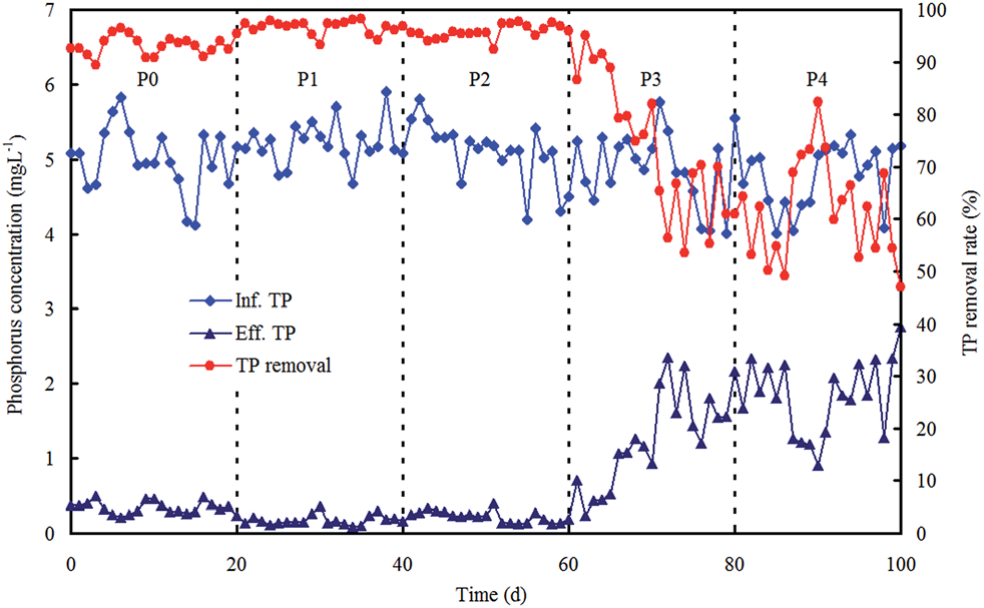
Reactor performance on phosphorus removal in each phase with various concentrations of ZnO NPs.

For phosphorus removal, TP removal rate was kept above 90% in phases P0, P1 and P2 (Fig. 3). This implies the negligible influence of the ZnO NPs on TP removal below a concentration of 0.8 mg L^−1^. When the concentration of ZnO NPs increased to 1.5 mg L^−1^ or higher, TP removal dramatically decreased. These results illustrate that the growth and activity of polyphosphate accumulating organisms (PAOs) are inhibited through the anaerobic release and aerobic uptake of phosphorus, thereby phosphorus removal clearly drops.^23,24^ However, COD removal was unaffected during the whole experimental period (data not shown). A previous study also reported that COD removal was not impacted adversely until the concentration of ZnO NPs reached as high as 68 mg L^−1^.^25^

### 3.2. Responses of the microbial community successional patterns

#### 3.2.1 Effects of ZnO NPs on microbial community diversities

To further understand the effect of ZnO NPs on the bacterial community diversity and richness in the reactor during long-term exposure, high-throughput sequencing was performed using an Illumina Miseq system. The Good’s coverage values of five samples were about 0.9, indicating that the bacterial communities could well represent the total sequences of the activated sludge samples. Based on a 97% similarity of sequences, the retrieved sequences were clustered into 1320, 1270, 1160, 1135 and 1363 operational taxonomic units (OTUs) at ZnO NP concentrations of 0, 0.25, 0.8, 1.5 and 4 mg L^−1^, respectively (Table 2).

**Table 2:** Sequencing results of the sludge sample in different phases

Sample	Sequence numbers	OTU number	Shannon index	Chao1 index	ACE index	Simpson index
P0	49 534	1320	10.30	6421.45	7117.18	0.997
P1	44 043	1270	10.09	6322.17	7250.18	0.997
P2	31 183	1160	9.62	6379.32	7452.33	0.995
P3	33 788	1135	9.33	5723.52	6530.43	0.994
P4	36 773	1363	9.84	5407.57	6428.06	0.997

The Shannon index, Simpson index, ACE index and Chao 1 index are commonly used for indicating the microbial diversity and richness.^26^ Compared with 0 mg L^−1^ ZnO NPs, the Shannon, Chao 1 and ACE indexes showed significant changes at 1.5 and 4 mg L^−1^ ZnO NPs (Table 2), suggesting that the presence of the ZnO NPs affected the microbial diversity and richness of the activated sludge in the OCO reactor.

#### 3.2.2 Successional changes to the microbial community under ZnO NP stress

**Fig. 4:**
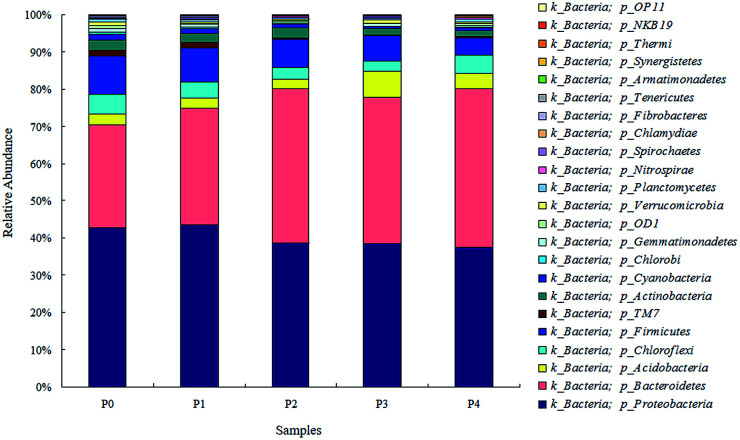
Phylum-level distribution of microbial communities in the reactor.

**Fig. 5:**
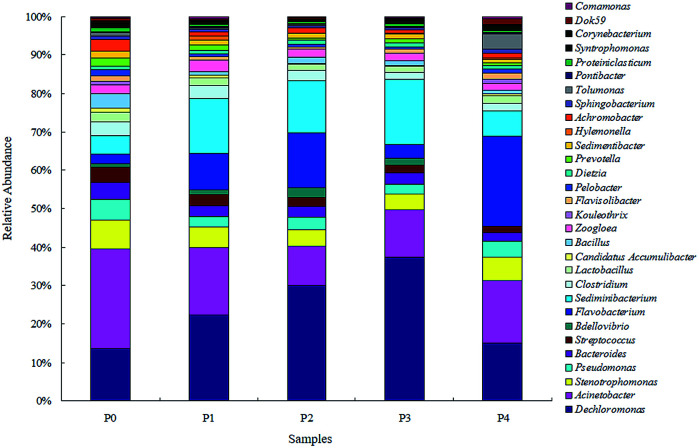
Taxonomic classification of sequences at bacterial genus level in the dominated classes.

As the microbial diversity and richness of the activated sludge were impacted by the ZnO NPs, the microbial community patterns at the phylum level for each phase are further illustrated in Fig. 4. The prominent phyla of the five sludge samples consisted of *Proteobacteria* (38.21–43.44%), *Bacteroidetes* (27.47–43.36%), *Firmicutes* (4.96–10.00%), *Chloroflexi* (2.95–5.30%), *Acidobacteria* (2.20–6.75%), *Actinobacteria* (1.60–2.59%), *Cyanobacteria* (0.61–1.67%) and TM7 (0.17–1.74%) at various ZnO NP concentrations. As the concentrations of the ZnO NPs increased from 0 mg L^−1^ to 4 mg L^−1^, the variation in the microbial phyla abundance showed that the proportions of *Proteobacteria*, *Firmicutes* and *Actinobacteria* decreased slightly, whereas those of *Bacteroidetes* and *Acidobacteria* increased. Previous studies reported that *Proteobacteria* and *Bacteroidetes* were the predominant phyla in most full-scale WWTPs,^27,28^ together with the subdominant phyla *Firmicutes* and *Acidobacteria*.^29^ Specifically, *Proteobacteria* could play a crucial role in the removal of organic waste and nutrients.^30,31^*Chloroflexi* are also common filamentous bacteria in the biological treatment processes of wastewater.^32^ At the genus level, *Acinetobacter*, *Stenotrophomonas*, *Pseudomonas* and *Bacteroides* decreased with increasing ZnO NP concentrations (Fig. 5). Previous studies showed that members of the genus *Acinetobacter* were typical bacteria present in sewage^33^ and versatile in the biodegradation of various pollutants,^34^ and *Stenotrophomonas* and *Pseudomonas* were classified as organophosphorus-degrading bacteria.^35^ On the other hand, *Dechloromonas* and *Sediminibacterium* firstly increased with increasing ZnO NP concentrations up to 1.5 mg L^−1^ (P0–P3), and decreased at the ZnO NP concentration of 4 mg L^−1^ (P4). Previous reports illustrated that *Dechloromonas* were denitrifiers^36^ or capable of reducing nitrate or nitrite to nitrogen gas under autotrophic conditions.^37^*Dechloromonas* also exhibited high tolerance to ZnO NPs in an SBR^38^ and an increasing tendency with elevated ZnO NP concentrations.^39^

**Fig. 6:**
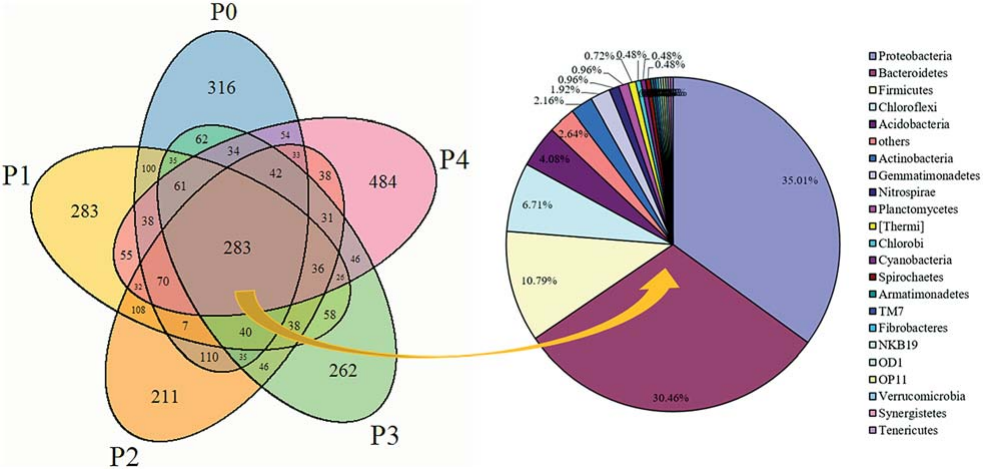
Venn diagrams based on high-throughput sequencing of the microbial community at the P1–P4 stages (OTU at 3% difference). The shared OTUs were analyzed at the phylum level.

Microbial similarities and differences of activated sludge samples were analyzed by a Venn diagram (Fig. 6). The total of observed OTUs in all five communities was 3074, but only 283 OTUs (9.2%) of the total OTUs were shared among them (Fig. 6). The shared OTUs in the five communities indicated that some microbes existed in the activated sludge during the whole operational period. Furthermore, the majority of the shared OTUs at the phyla level were *Proteobacteria* (35.01%) and *Bacteroidetes* (30.46%), followed by *Firmicutes* (10.79%), *Chloroflexi* (6.71%) and *Acidobacteria* (2.64%). The number of OTUs that were unique to each community were counted at 316 (P0), 283 (P1), 211 (P2), 262 (P3) and 484 (P4), and together they accounted for 90.8% of the total number of observed OTUs, suggesting that ZnO NPs altered the microbial community compositions with long-term exposure.

## 4. Conclusions

The long-term exposure of low-level ZnO NPs caused adverse impacts on the removal of nitrogen and phosphorus in an integrated OCO reactor. ZnO NPs resulted in the chronic decline of microbial diversity and richness. The shifts in the microbial communities revealed that *Bacteroidetes* and *Proteobacteria* were the predominant phyla, and even some common taxa existing in the activated sludge all the time experienced dramatic variations in the composition of the microbial species. The present results are significant in understanding the functional performance and microbial community successions of activated sludge under much lower ZnO NP stress.

## Conflicts of interest

There are no conflicts to declare.

## Supplementary Material

## References

[cit1] Yang Y., Zhang C., Hu Z. (2013). Environ. Sci.: Processes Impacts.

[cit2] Nel A., Xia T., Mädler L., Li N. (2006). Science.

[cit3] Gottschalk F., Sonderer T., Scholz R. W., Nowack B. (2009). Environ. Sci. Technol..

[cit4] Sun T. Y., Gottschalk F., Hungerbühler K., Nowack B. (2014). Environ. Pollut..

[cit5] Li M., Zhu L., Lin D. (2011). Environ. Sci. Technol..

[cit6] Chen Q., Li T., Gui M., Liu S., Zheng M., Ni J. (2017). Bioresour. Technol..

[cit7] Wang Z., Huang F., Mei X., Wang Q., Song H., Zhu C., Wu Z. (2014). J. Membr. Sci..

[cit8] Zhang X., Zhang N., Fu H., Chen T., Liu S., Zheng S., Zhang J. (2017). Bioresour. Technol..

[cit9] Tan M., Qiu G., Ting Y.-P. (2015). Bioresour. Technol..

[cit10] Puay N.-Q., Qiu G., Ting Y.-P. (2015). J. Cleaner Prod..

[cit11] Metch J. W., Burrows N. D., Murphy C. J., Pruden A., Vikesland P. J. (2018). Nat. Nanotechnol..

[cit12] Li D., Mao Y., Liu Z., Yin X., Lang C., Liu Y. (2014). Environ. Technol..

[cit13] Liu Z., Zhou H., Li D., Yin X., Mao Y., Chen W. (2017). Technol. Water Treat..

[cit14] APHA , Standard Methods for the Examination of Water and Wastewater, American Public Health Association/American Water Works Association/Water Environment Federation, Washington, DC, 2005

[cit15] Caporaso J. G., Kuczynski J., Stombaugh J., Bittinger K., Bushman F. D., Costello E. K., Fierer N., Pena A. G., Goodrich J. K., Gordon J. I., Huttley G. A., Kelley S. T., Knights D., Koenig J. E., Ley R. E., Lozupone C. A., McDonald D., Muegge B. D., Pirrung M., Reeder J., Sevinsky J. R., Tumbaugh P. J., Walters W. A., Widmann J., Yatsunenko T., Zaneveld J., Knight R. (2010). Nat. Methods.

[cit16] Edgar R. C., Haas B. J., Clemente J. C., Quince C., Knight R. (2011). Bioinformatics.

[cit17] Wang Q., Garrity G. M., Tiedje J. M., Cole J. R. (2007). Appl. Environ. Microbiol..

[cit18] Lu L., Xing D., Ren N. (2012). Water Res..

[cit19] Zhang D. Q., Eng C. Y., Stuckey D. C., Zhou Y. (2017). Chemosphere.

[cit20] Daverey A., Chen Y.-C., Sung S., Lin J.-G. (2014). Bioresour. Technol..

[cit21] Hou L., Xia J., Li K., Chen J., Wu X., Li X. (2013). Water Sci. Technol..

[cit22] Wang S. T., Li S. P., Wang W. Q., You H. (2015). RSC Adv..

[cit23] Lombi E., Donner E., Tavakkoli E., Turney T. W., Naidu R., Miller B. W., Scheckel K. G. (2012). Environ. Sci. Technol..

[cit24] Liu G., Wang D., Wang J., Mendoza C. (2011). Sci. Total Environ..

[cit25] Huang F., Wang Z., Mei X., Wu Z. (2013). Technol. Water Treat..

[cit26] Meli K., Kamika I., Keshri J., Momba M. N. B. (2016). Sci. Rep..

[cit27] Hu M., Wang X., Wen X., Xia Y. (2012). Bioresour. Technol..

[cit28] Shu D., He Y., Yue H., Wang Q. (2015). Bioresour. Technol..

[cit29] Xia S., Duan L., Song Y., Li J., Piceno Y. M., Andersen G. L., Alvarez-Cohen L., Moreno-Andrade I., Huang C.-L., Hermanowicz S. W. (2010). Environ. Sci. Technol..

[cit30] Jeon C. O., Lee D. S., Park J. M. (2003). Water Res..

[cit31] Yang Y., Quensen J., Mathieu J., Wang Q., Wang J., Li M., Tiedje J. M., Alvarez P. J. J. (2014). Water Res..

[cit32] Björnsson L., Hugenholtz P., Tyson G. W., Blackall L. L. (2002). Microbiology.

[cit33] Snaidr J., Amann R., Huber I., Ludwig W., Schleifer K. H. (1997). Appl. Environ. Microbiol..

[cit34] Ishii S. i., Koki J., Unno H., Hori K. (2004). Appl. Environ. Microbiol..

[cit35] Singh B. K. (2009). Nat. Rev. Microbiol..

[cit36] Heylen K., Vanparys B., Wittebolle L., Verstraete W., Boon N., De Vos P. (2006). Appl. Environ. Microbiol..

[cit37] Ma J., Wang Z., He D., Li Y., Wu Z. (2015). Water Res..

[cit38] Liu Z., Zhou H., Liu J., Yin X., Mao Y., Liu Z., Li Z., Xie W. (2016). RSC Adv..

[cit39] Wang S., Gao M., She Z., Zheng D., Jin C., Guo L., Zhao Y., Li Z., Wang X. (2016). Bioresour. Technol..

